# Early childhood health in Bielefeld, Germany (BaBi study): study protocol of a social-epidemiological birth cohort

**DOI:** 10.1136/bmjopen-2017-018398

**Published:** 2017-08-21

**Authors:** Jacob Spallek, Angelique Grosser, Chantal Höller-Holtrichter, Ina-Merle Doyle, Jürgen Breckenkamp, Oliver Razum

**Affiliations:** 1 Department of Public Health, Brandenburg University of Technology, Senftenberg, Germany; 2 Department of Epidemiology and International Public Health, School of Public Health, Bielefeld University, Bielefeld, Germany

**Keywords:** epidemiology, public health, cohort study

## Abstract

**Introduction:**

The heterogeneity among the German population is increasing. Sociodemographic differentials (eg, in education and migrant status) have been associated with health disparities. Life course studies show that a considerable part of these disparities is determined by exposures during pregnancy and early childhood. The BaBi study was established in 2012 to investigate the production of health disparities from foetal life to childhood in the city of Bielefeld, Germany.

**Methods and analysis:**

Between 2013 and 2016, detailed information on socioeconomic characteristics, migration background, lifestyle factors, environmental factors, healthcare use, and health status of 995 newborns, including 24 twins, and their families was collected using standardised instruments. Data collection started during pregnancy or shortly after birth with a computer-assisted personal interview of the pregnant woman/mother. Follow-up data will be collected until 2018 by computer-assisted telephone interviews around the first, second and after the third birthday of the child and by linking routine healthcare data. Blood samples are collected from a small subsample of 50 mothers for a substudy about stress during pregnancy (BaBi-Stress study).

**Ethics and dissemination:**

The study was approved by the ethical committee of the Medical Faculty of Muenster University and the Data Protection Board of Bielefeld University. Results will be published in scientific journals. Data sets and questionnaires will be made accessible for researchers based on access proposals and data usage contracts.

**Strengths and limitations of this study**

- The BaBi birth cohort study is the first birth cohort in Germany with an explicit social-epidemiological perspective and will help analyse the production of health inequalities among newborns and children based on longitudinal data with a focus on social and cultural determinants of health.
- The findings will help to develop new explanatory models for health inequalities and to design specific interventions to improve health of newborns, in particular of socially deprived or immigrant families.
- The BaBi study faces similar risks of bias as other population-based, observational birth cohorts do. As the study relies on voluntary participation of pregnant women, bias due to self-selection of participants cannot be ruled out.
- The BaBi study has implemented various measures to recruit and follow-up all newborns in the city of Bielefeld, independent of their social or cultural background. Nevertheless, the response among lower educated families or families with a migrant background is lower than those among higher educated families or without a migrant background.

## Introduction

The heterogeneity of the population in Germany is increasing. Sociodemographic differentials (eg, in education and migrant status) have been associated with health disparities. Therefore, in order to adequately describe public health in Germany, epidemiological studies need to enrol participants from all population groups.^1^

Although Germany has a healthcare system that should pose no legal or financial barriers to subgroups of the population, considerable health inequalities exist. Differences arise from different health behaviours, exposures, environments, genes, and so on.^2 3^

Life course approaches show that a considerable part of these inequalities is determined by exposures, health status and development *in utero* and early childhood.^4–6^ Moreover, in early childhood, children are particularly vulnerable to the influence of social factors, for example, exposures due to poor living conditions. Turning this into a research perspective for social epidemiology, the health trajectory in early childhood is a valuable indicator for (1) the influence of social factors on the health situation, and (2) the health opportunities in later life. Social factors contributing to the production of health differentials act on the individual and the contextual level and start influencing health *in utero* and soon after birth.

While the fact as such is well documented, underlying mechanisms remain unclear. It is still poorly understood how specific social factors like ethnicity and migration history, socioeconomic status and living conditions act in creating health inequalities among children. Moreover, interactions between these factors need to be investigated. There is a need to identify and quantify the causes of health inequalities in childhood, looking beyond parental factors or the socioeconomic situation of the parents.

The overall aim of the BaBi study (“*Gesundheit von Babys und Kindern in Bielefeld*”) is to improve understanding of the health trajectory of children, with a focus on the increasing heterogeneity among the German population due to social determinants, cultural diversity and migration background. The findings will add important evidence in the shape of social-epidemiological data for the development of (1) new explanatory models of health inequalities, and (2) specific prevention approaches and interventions to improve the health status of socially deprived children and those with a migration background. In particular, emphasis was put on recruiting the offspring of families with a migration background. This posed a methodological challenge as tailored measures are necessary to increase participation of populations with diverse migration background in epidemiological studies.^7 8^

The project focuses on four primary areas: (1) pre/postnatal growth, physical development; (2) mental and cognitive development; (3) allergic diseases in childhood; (4) access to, and utilisation of, healthcare among pregnant women and children (antenatal care and the standard childhood screening examinations).

## Methods and analysis

The BaBi study is a prospective, population-based cohort study of newborns in Bielefeld, Germany. The aim was to recruit a total of 1500 newborns from October 2013 to October 2015. A special focus was laid on the inclusion of women with migration background (who either migrated themselves or are the offspring of immigrants), as this population group has not been properly represented in German birth cohorts so far. Due to an initially low participation rate, recruitment strategies were adapted and the recruitment period extended to December 2016, resulting in a total of 995 recruited newborns, including 24 twins.

Women were enrolled either during pregnancy or within 8 weeks after birth. Recruitment initially aimed to reach women during pregnancy via gynaecologists and midwives who provided basic information about the study along with a flyer and information sheet. With this strategy, however, response rates were low for women of lower social status and women with a migration background. Furthermore, it proved time consuming and thus impractical for the gynaecologists and midwives. Therefore, several adaptations to the recruitment approach were made in 2014. First, our project staff or clinic staff approached potential participants in all obstetric hospitals in Bielefeld. Second, we extended the recruitment period until 8 weeks after birth. Third, we provided gynaecologists and midwives with an information package (consisting of a study flyer, a detailed information sheet and a postpaid response envelope) which they handed over to pregnant women. This revised strategy was supported by an increase in public relation work in the city of Bielefeld. As a consequence, women contacted by the study team in obstetric hospitals usually had already heard about the study. Response subsequently improved, in particular among hard-to-reach women.

### Follow-up and outcome measures

**Figure 1:**
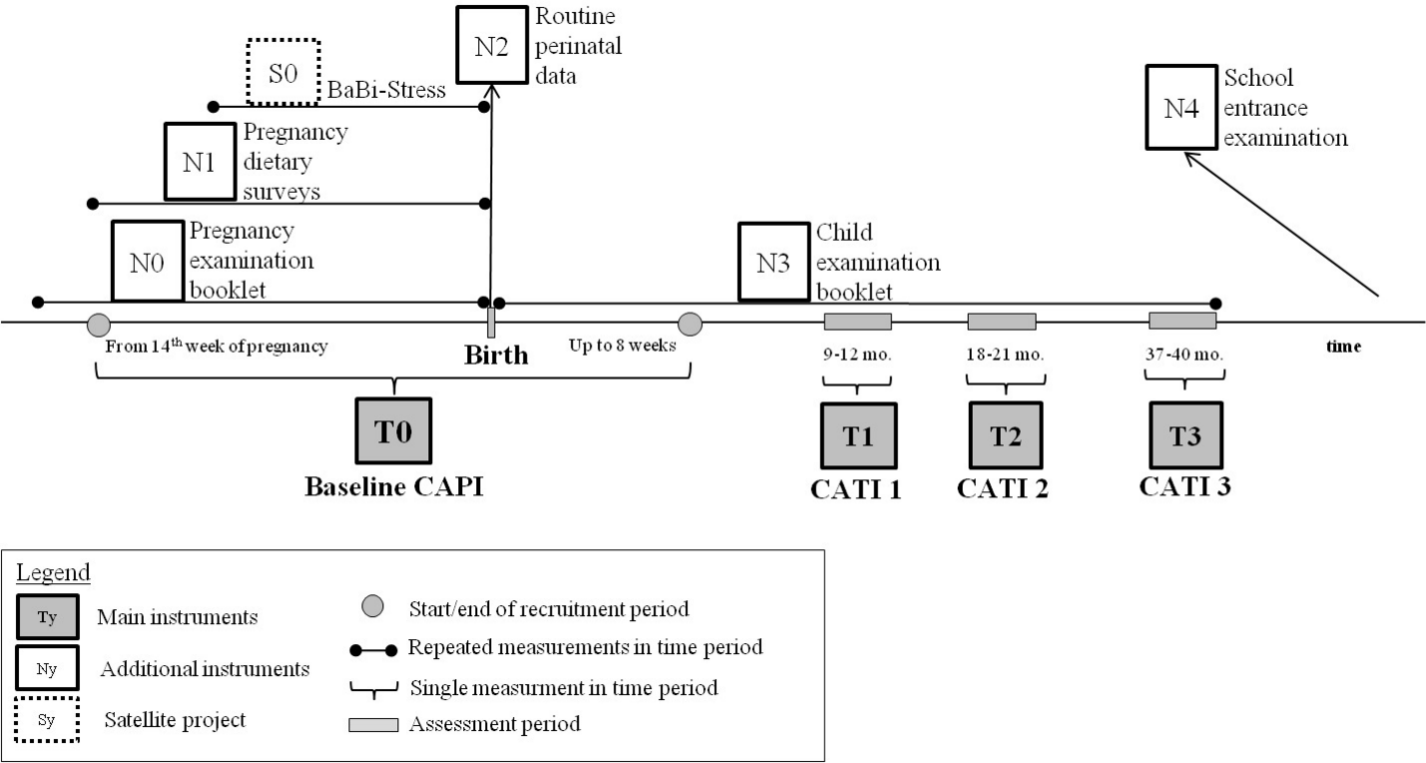
Data collection and follow-up in the BaBi study. CAPI, computer-assisted personal interview; CATI, computer-assisted telephone interview.

The primary outcomes of the study are measures of health of the newborns/children. Respective data have been collected from pregnancy or birth; data collection will continue until the age of 4 years (see figure 1). One computer-assisted personal interview (CAPI) is conducted at recruitment, followed by three computer-assisted telephone interviews (CATI) in the first 40 months. Additionally, data routinely collected during pregnancy (antenatal care booklet), at birth (perinatal hospital data) and in the child health examination booklets of the eight standard early childhood health examinations (in German: *U-Untersuchungen*) U1–U8, as well as contextual data from the city of Bielefeld are linked to the interview data. At age 5–6 years, data from the routine school entry examination can be linked as well. Health outcomes of children aged 0–4 years are extracted covering three main fields: (1) physical development (weight gain, birth weight, weight trajectory), (2) mental/cognitive development and (3) nutrition and allergies. The final data set also (4) contains process indicators such as uptake of antenatal care and of early childhood examinations. Table 1 provides an overview of data sources and collection at the different follow-up stages.

**Table 1:** Overview of outcome measures collected, by follow-up stage

Phase	Measurements
Baseline CAPI	Demographic and socioeconomic status, pregnancy details, nutrition, body image, health behaviour, breastfeeding intentions, utilisation of non-medical antenatal care, well-being, stress/anxieties, living and social environment, psychological profile, acculturation and language, religion, information about partner (eg, health, partnership)
Pregnancy diet survey (telephone based)	Brief 24-hour food frequency questionnaire, consumption of food groups
Pregnancy examination booklet	Gestational weight gain, pregnancy risks, medical problems in pregnancy, growth of fetus, antenatal care use
Routine perinatal data	Birth outcomes (eg, length and weight of newborn), type of delivery, utilisation of medical antenatal care
CATI 1	Experiences with infant feeding and breast feeding/breast milk substitutes, infant diet (eg, drinks, food, allergies, problems, worries)
CATI 2	Employment and child care arrangements, pet ownership, child health, sleep, language development and spoken language at home, media use
CATI 3	Demographic and socioeconomic status, living and social environment of child, child health, physical and psychological development, allergies, leisure time activities, eating routine
Child examination booklet	Physical and cognitive development of child at different time points, use of medical service
Ongoing	All participants are flagged to allow later linkage with routine data from school entrance examination

CAPI, computer-assisted personal interview; CATI, computer-assisted telephone interview.

**Figure 2:**
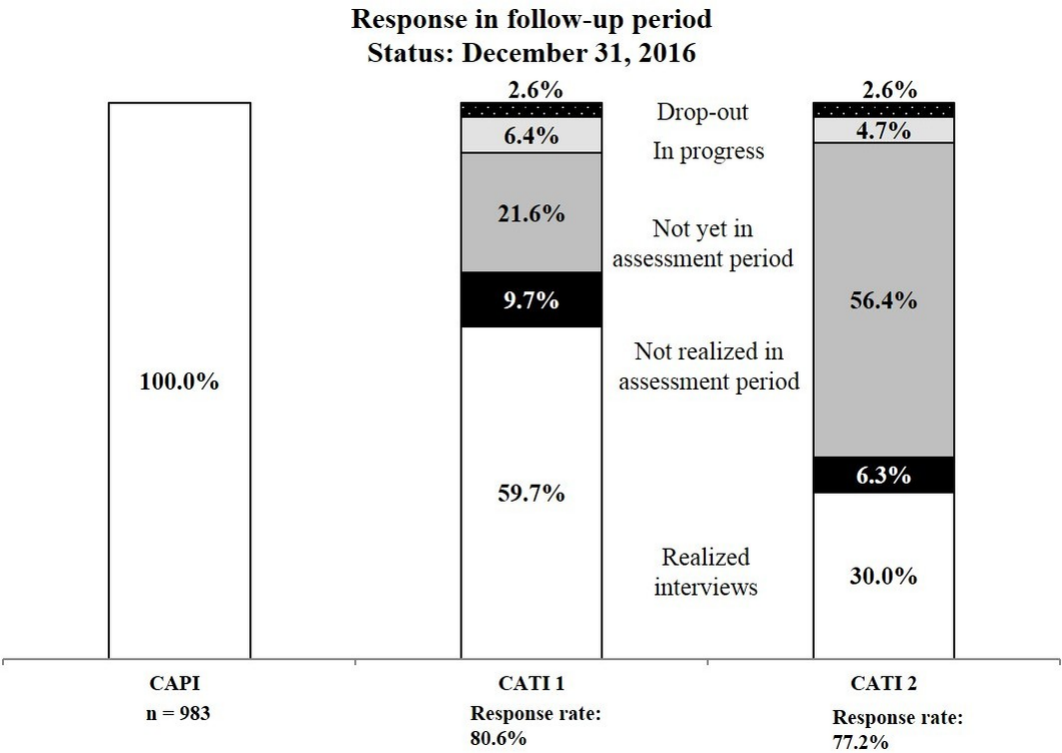
Response in follow-up period. CAPI, computer-assisted personal interview; CATI, computer-assisted telephone interview.

In 2016, the first children enrolled in the study reached the age of 3 years, response rates at follow-ups 1 and 2 are shown in figure 2, based on 983 CAPIs conducted with the mothers (the number of newborns was 995, as there were 24 twin births). As CATI 3 has started only recently, no data on response are available yet.

Blood samples were collected from a subsample of 50 pregnant women for a satellite study on stress during pregnancy (BaBi-Stress study, funded by the *Deutsche Forschungsgemeinschaft*). This study focuses on measures of alterations in endocrine (salivary cortisol, placental corticotropin releasing hormone (CRH) and immune/inflammatory (C-reactive protein, interleukin-6) stress biology over gestation as the primary outcome of interest and a proximate pathway of transmission of the effects of maternal migration-related experiences on foetal development and offspring disease risk.

### Analysis

Recruitment has now been completed. In line with the aims of the study, univariate and multivariate analyses regarding the influence of individual and contextual exposures on outcome and process variables such as antenatal care uptake are ongoing. Further data analyses are planned to gain a deeper understanding of the influence of individual and contextual exposures on health inequalities and the trajectory of health in early childhood. So far, two systematic reviews and a paper describing the study aims have been published.^9–11^ Methodological papers on the recruitment of migrants for birth cohort studies in Germany and on data linkage of primary and secondary data using advanced statistical procedures were submitted or are in progress. Preliminary data analyses have been conducted regarding the determinants of antenatal stress and dietary patterns in pregnancy.

## Discussion

The BaBi birth cohort study will help analyse the production of health inequalities among newborns and children in a German city based on longitudinal data with a focus on social and cultural determinants of health. So far, the BaBi study is the only birth cohort in Germany with an explicit social-epidemiological perspective. The findings will help develop new, or revise existing, explanatory models for health inequalities and develop specific interventions to improve health of newborns, in particular of socially deprived or immigrant families.

The BaBi study faces similar risks of bias as other population-based, observational birth cohorts do. As the study relies on voluntary participation of pregnant women, bias due to self-selection of participants cannot be ruled out completely. However, the BaBi study has implemented various measures, for example, in the shape of culturally sensitive recruitment and public relations, to recruit and follow-up all newborns in the city of Bielefeld, independent of their social or cultural background. Nevertheless, the response rates among lower educated families or families with a migrant background are lower than those among higher educated families or without a migrant background.

### Ethics and dissemination

The study was approved by the ethics committee of the Medical Faculty of Muenster University and the Data Protection Board of Bielefeld University. Data are pseudonymised and all analyses are conducted with fully anonymised data sets. Data and questionnaires will be made accessible to researchers based on an access proposal which will be assessed by the BaBi Executive Committee. Researchers of positively evaluated proposals will be provided the data and questionnaire for a specified time period after signing a data usage contract.

## Supplementary Material

Reviewer comments

Author's manuscript
