# Supplementary material for: Early childhood health in Bielefeld, Germany (BaBi study): study protocol of a social-epidemiological birth cohort
Source: BMJ Open. 2017 Aug 21;7(8):e018398. doi: 10.1136/bmjopen-2017-018398 (PMC5724168; doi:10.1136/bmjopen-2017-018398)
Supplement: Author's manuscript [file bmjopen-2017-018398.draft_revisions.pdf]

PEER REVIEW HISTORY

BMJ Open publishes all reviews undertaken for accepted manuscripts. Reviewers are asked to complete a checklist review form (**http://bmjopen.bmj.com/site/about/resources/checklist.pdf**) and are provided with free text boxes to elaborate on their assessment. These free text comments are reproduced below.

ARTICLE DETAILS

**TITLE (PROVISIONAL)**	Early childhood health in Bielefeld, Germany (BaBi study): study protocol of a social-epidemiological birth cohort
**AUTHORS**	Spallek, Jacob; Grosser, Angelique; Höller-Holtrichter, Chantal; Doyle, Ina; Breckenkamp, Jürgen; Razum, Oliver

This article was not externally reviewed at BMJ Open. Protocols that have been independently assessed prior to submission to BMJ Open are usually fast-tracked to publication on the grounds that further substantial changes will not be possible. This independent assessment will usually be external, independent review for both a substantial grant award from a non-commercial or government funder and ethics approval.
